# Supplementary material for: A temperature-induced metabolic shift in the emerging human pathogen Photorhabdus asymbiotica
Source: mSystems. 2024 Oct 24;9(11):e00970-23. doi: 10.1128/msystems.00970-23 (PMC11575385; doi:10.1128/msystems.00970-23)
Supplement: File S1 — BLASTp ortholog search. [file msystems.00970-23-s0001.docx]

**Supplementary File 1**

Reactions in the draft metabolic reconstruction for *Photorhabdus asymbiotica*, obtained from the Model SEED, which had an unknown gene-protein-reaction association. These reactions were searched in the BiGG database and genes associated with these reactions in candidate prokaryotic reconstructions were used in a BLASTp orthology search to identify orthologous genes in *P. asymbiotica* and identify the gene-protein-reaction association in the reconstruction.

| **Reaction** | ***E. coli* str. K-12 substr. MG1655** | ***S. aureus* subsp. aureus USA300_TCH1516** | ***M. barkeri* str. Fusaro** | ***G. metallireducens* GS-15** | ***E. coli* APEC O1** | ***C. difficile* 630** | ***E. coli* BL21(DE3)** | ***B. subtilis* subsp. Subtilis str. 168** | ***A. baumannii* AYE** | **Orthologous Gene in *P. asymbiotica*** |
| --- | --- | --- | --- | --- | --- | --- | --- | --- | --- | --- |
| FEROc - Ferroxidase | - | USA300HOU_RS11550 | - | - | - | - | - | - | - | PAU_RS14185 |
| HMBS - Hydroxymethylbilane synthase | b3805 | - | Mbar_A1465 | Gmet_3234 | APECO1_RS21655 | - | B21_03629 | - | - | PAU_RS20480 |
| GLUSy - Glutamate synthase (NADPH) | b3212 and b3213 | - | - | - | APECO1_RS18510 and APECO1_RS18515 | CD630_13390 or CD630_15370 | B21_03028 and B21_03029 | - | - | PAU_RS18085 and PAU_RS18090 |
| AHCi - Adenosylhomocysteinase | - | - | Mbar_A3251 | Gmet_1294 | - | CD630_26110 | - | - | - | PAU_RS13775 |
| ACALD - Acetaldehyde dehydrogenase (acetylating) | b1241 or b0351 | - | - | - | APECO1_RS06570 | CD630_03340 or CD630_19170 or CD630_29660 | B21_00309 or B21_01225 | - | - | PAU_RS09815 |
| PTAr - Phosphotransacetylase | b2297 or b2458 | - | Mbar_A1821 | Gmet_1035 | APECO1_RS13755 | - | B21_02182 or B21_02311 | - | - | PAU_RS07390 |
| PPGPPDP - Guanosine-3',5'-bis(diphosphate) 3'-diphosphatase | b3650 | - | - | Gmet_2399 | APECO1_RS20845 | CD630_27440 or CD630_03450 | B21_03459 | - | - | PAU_RS01035 |
| MDH - Malate dehydrogenase | b3236 | - | Mbar_A0309 | Gmet_1360 | APECO1_RS18590 | - | B21_03047 | - | - | PAU_RS20085 |
| SUCD1 - Succinate dehydrogenase | b0722 and b0721 and b0723 and b0724 | - | - | - | - | - | - | - | - | PAU_RS1465 |
| ASPK - Aspartate kinase | b3940 or b0002 or b4024 | - | Mbar_A0861 | Gmet_1880 | APECO1_RS00010 or APECO1_RS22465 or APECO1_RS22975 | CD630_13220 or CD630_20540 or CD630_26270 | B21_00002 or B21_03775 or B21_03856 | - | - | PAU_RS21135 or PAU_RS19520 |
| FDH - Formate dehydrogenase | - | USA300HOU_RS00935 | Mbar_A1562 and Mbar_A1561 | - | - | CD630_33170 and CD630_2179 and CD630_0769 | - | - | - | PAU_RS21830 |
| ARGDC - Arginine decarboxylase | b4117 | USA300HOU_RS02450 | Mbar_A2039 or Mbar_A2492 | Gmet_0904 | APECO1_RS23565 | CD630_08880 or CD630_35510 | B21_03949 | - | - | PAU_RS06445 |
| PHETA1 - Phenylalanine transaminase | b3770 or b4054 or b0928 | (USA300HOU_RS14535 and USA300HOU_RS03910) or USA300HOU_RS10515 | Mbar_A3133 or Mbar_A2083 or Mbar_A3463 | Gmet_3325 or Gmet_3433 or Gmet_0732 | APECO1_RS23155 or APECO1_RS04750 or APECO1_RS21480 | CD630_01070 or CD630_13390 or CD630_28280 or CD630_15490 or CD630_22000 | B21_03886 or B21_00939 or B21_03597 | - | - | PAU_RS20690 or PAU_RS19410 or PAU_RS13445 |
| TYRTA - Tyrosine transaminase | b0928 or b4054 | (USA300HOU_RS14535 and USA300HOU_RS03910) or USA300HOU_RS10515 | Mbar_A2083 or Mbar_A3463 or Mbar_A3133 | Gmet_3325 or Gmet_3433 or Gmet_0732 | APECO1_RS23155 or APECO1_RS04750 | CD630_01070 or CD630_13390 or CD630_28280 or CD630_15490 or CD630_22000 | B21_03886 or B21_00939 | - | - | PAU_RS13445 or PAU_RS19410 |
| ALCD2x - Alcohol dehydrogenase (ethanol) | b1478 or b0356 or b1241 | - | - | Gmet_1046 or Gmet_1053 | APECO1_RS08185 or APECO1_RS06570 or APECO1_RS01845 | CD630_03340 or CD630_30060 or CD630_29660 | B21_01448 or B21_01225 or B21_00314 | - | - | PAU_RS09815 |
| GLYK - Glycerol kinase | b3926 | - | - | Gmet_1676 | APECO1_RS22395 | - | B21_03760 | - | - | PAU_RS21200 |
| AMPTASECG - Alanyl aminopeptidase (cys-gly) | b0932 or b0237 or b2523 or b4260 | USA300HOU_RS08170 | - | - | APECO1_RS01350 or APECO1_RS04775 or APECO1_RS24725 or APECO1_RS14315 | CD630_07080 or CD630_13000 | B21_00235 or B21_00943 or B21_04090 or B21_02379 | - | - | PAU_RS13420 or PAU_RS15980 or PAU_RS06660 or PAU_RS19770 |
| PTA2 - Phosphate acetyltransferase | b2297 | - | - | Gmet_1035 | - | - | B21_02182 | - | - | PAU_RS07390 |
| RPI - Ribose-5-phosphate isomerase | b2914 or b4090 | USA300HOU_RS12640 | Mbar_A3172 | Gmet_2621 or Gmet_1604 | APECO1_RS16520 or APECO1_RS23430 | CD630_23200 and CD630_34800 | B21_02708 or B21_03922 | BSU36920 | - | PAU_RS05635 |
| INSK - Insosine kinase | b0477 | - | - | - | APECO1_RS02425 | - | B21_00433 | - | - | PAU_RS16770 |
| ADSS - Adenylosuccinate synthase | b4177 | USA300HOU_RS00085 | Mbar_A0403 | Gmet_3260 | APECO1_RS24290 | CD630_36550 | B21_04006 | BSU40420 | - | PAU_RS20230 |
| ALAALAr - D-alanine-D-alanine ligase (reversible) | b0092 or b0381 | USA300HOU_RS11245 | - | Gmet_0415 | APECO1_RS01940 or APECO1_RS00500 | CD630_14080 | B21_00332 or B21_00092 | BSU04560 | - | PAU_RS05465 |
| GSNK - Guanosine kinase | b0477 | - | - | - | APECO1_RS02425 | - | B21_00433 | - | - | PAU_RS16770 |
| CYSTL - Cystathionine b-lyase | b3008 or b1622 | USA300HOU_RS01900 | - | Gmet_0698 or Gmet_0699 | APECO1_RS08705 or APECO1_RS17465 | CD630_30290 or CD630_24840 or CD630_26960 or CD630_27330 | B21_01581 or B21_02831 | BSU11880 | - | PAU_RS17395 or PAU_RS14860 |
| ASPCT - Aspartate carbamoyltransferase | b4245 and b4244 | - | Mbar_A1168 | Gmet_1769 | (APECO1_RS24650 and APECO1_RS24655) or APECO1_RS24655 | CD630_35880 | (B21_04076 and B21_04077) or B21_04077 | BSU15490 | - | PAU_RS19845 |
| GLYCK - Glycerate kinase | b0514 | - | - | - | APECO1_RS02610 | CD630_30840 | B21_00469 | BSU40040 | - | PAU_RS18465 |
| A5PISO - Arabinose-5-phosphate isomerase | b2708 or b3197 | - | - | Gmet_1278 | APECO1_RS18435 or APECO1_RS15415 | - | B21_03013 or B21_02523 | - | - | PAU_RS18215 |
| DMATT - Dimethylallyltranstransferase | b0421 | USA300HOU_RS08130 | Mbar_A1417 | Gmet_1935 or Gmet_2455 | APECO1_RS02140 | CD630_12050 | B21_00373 | BSU24280 | - | PAU_RS17030 |
| PPND - Prephenate dehydrogenase | b2600 | USA300HOU_RS06950 | Mbar_A0924 | Gmet_0863 | APECO1_RS14730 | CD630_18390 | B21_02453 | BSU22610 | - | PAU_RS15835 |
| PPND2 - Prephenate dehydrogenase NADP | - | USA300HOU_RS06950 | - | - | - | - | - | - | - | PAU_RS15835 |
| HSDy - Homoserine dehydrogenase (NADPH) | b3940 or b0002 | USA300HOU_RS06750 | Mbar_A1898 | Gmet_1629 | APECO1_RS22465 or APECO1_RS00010 | CD630_11660 or CD630_15800 | B21_03775 or B21_00002 | - | - | PAU_RS21135 or PAU_RS02630 |
| DDPA - 3-deoxy-D-arabino-heptulosonate 7-phosphate synthetase | b0754 or b2601 or b1704 | - | - | Gmet_0024 or Gmet_0346 or Gmet_2375 | APECO1_RS14735 or APECO1_RS03640 or APECO1_RS09150 | CD630_14530 or CD630_18320 | - | BSU29750 | - | PAU_RS14375 or PAU_RS15840 or PAU_RS09160 |
| HACD1_1 - 3 hydroxyacyl CoA dehydrogenase acetoacetyl CoA | - | - | - | - | - | - | - | BSU32840 or BSU24160 | - | PAU_RS11830 |
| GRTT - Geranyltranstransferase | b0421 | USA300HOU_RS08130 | Mbar_A1417 or Mbar_A1074 or Mbar_A2346 or Mbar_A0263 | Gmet_1935 or Gmet_2455 | APECO1_RS02140 | - | B21_00373 | BSU24280 | - | PAU_RS17030 |
| G1PTT - Glucose-1-phosphate thymidylyltransferase | b3789 or b2039 | - | Mbar_A0233 | Gmet_0924 | APECO1_RS11320 or APECO1_RS21570 | - | B21_03616 | - | - | PAU_RS20560 |
| DHQTi - 3-dehydroquinate dehydratase, irreversible | b1693 | USA300HOU_RS04355 | Mbar_A0922 | Gmet_0981 | APECO1_RS09085 | - | B21_01651 | BSU23080 | - | PAU_RS18365 |
| UPP3S - Uroporphyrinogen-III synthase | b3804 | USA300HOU_RS08840 | Mbar_A1792 | Gmet_3235 | APECO1_RS21650 | CD630_34200 | B21_03628 | BSU28140 | - | PAU_RS20485 |
| UAMAS - UDP-N-acetylmuramoyl-L-alanine synthetase | b0091 | USA300HOU_RS09245 | - | Gmet_0413 | APECO1_RS00495 | CD630_35180 | B21_00091 | BSU29790 | - | PAU_RS05460 |
| TMPPP - Thiamine-phosphate diphosphorylase | b3993 | USA300HOU_RS11295 | Mbar_A3318 | Gmet_2909 | APECO1_RS22775 | CD630_16010 or CD630_17060 | B21_03823 | BSU38290 | - | PAU_RS01940 |
| PFK_2 - Phosphofructokinase | b3916 | - | - | - | APECO1_RS22340 | CD630_34510 | B21_03750 | BSU29190 | - | PAU_RS21235 |
| SHCHD2 - Sirohydrochlorin dehydrogenase (NAD) | b3368 | USA300HOU_RS12965 | Mbar_A1461 and Mbar_A1791 | Gmet_3231 | APECO1_RS19325 | CD630_34180 | B21_03171 | - | - | PAU_RS03400 |
| HACD9 - 3 hydroxyacyl CoA dehydrogenase 2 Methylacetoacetyl CoA | - | - | - | - | - | CD630_11820 | - | BSU32840 | - | PAU_RS08255 |
| MHPGLUT - 5 methyltetrahydropteroyltriglutamate homocysteine S methyltransferase | - | USA300HOU_RS01890 | - | - | - | - | - | - | - | PAU_RS19295 |
| UM4PL - UDP-N-acetylmuramate:L-alanyl-gamma-D-glutamyl-meso-diaminopimelate-D-alanine ligase | b4233 | - | - | - | APECO1_RS24590 | - | B21_04065 | - | - | PAU_RS20095 |
| MOAT - 3-deoxy-D-manno-octulosonic acid transferase | b3633 | - | - | Gmet_2348 | APECO1_RS20760 | - | B21_03442 | - | - | PAU_RS21685 |
| HACD7i - 3-hydroxyacyl-CoA dehydrogenase (3-oxohexadecanoyl-CoA) | b3846 or b2341 | - | - | - | APECO1_RS21985 or APECO1_RS12990 | - | B21_03686 or B21_02226 | - | - | PAU_RS19205 or PAU_RS06925 |
| HACD5i - 3-hydroxyacyl-CoA dehydrogenase (3-oxododecanoyl-CoA) | b2341 or b3846 | - | - | - | APECO1_RS21985 or APECO1_RS12990 | - | B21_03686 or B21_02226 | - | - | PAU_RS19205 or PAU_RS06925 |
| HACD4i - 3-hydroxyacyl-CoA dehydrogenase (3-oxodecanoyl-CoA) | b2341 or b3846 | - | - | - | APECO1_RS21985 or APECO1_RS12990 | - | B21_03686 or B21_02226 | - | - | PAU_RS19205 or PAU_RS06925 |
| HACD3i - 3-hydroxyacyl-CoA dehydrogenase (3-oxooctanoyl-CoA) | b3846 or b2341 | - | - | - | APECO1_RS12990 or APECO1_RS21985 | - | B21_02226 or B21_03686 | - | - | PAU_RS19205 or PAU_RS06925 |
| HACD2i - 3-hydroxyacyl-CoA dehydrogenase (3-oxohexanoyl-CoA) | b2341 or b3846 | - | - | - | APECO1_RS21985 or APECO1_RS12990 | - | B21_03686 or B21_02226 | - | - | PAU_RS19205 or PAU_RS06925 |
| URFGTT - UDP-L-rhamnose:flavonol-3-O-D-glucoside L-rhamnosyltransferase | - | - | - | - | APECO1_RS21925 | - | - | - | ABAYE_RS17020 | PAU_RS19260 |
| MOAT2 - 3-deoxy-D-manno-octulosonic acid transferase | b3633 | - | - | Gmet_2348 | APECO1_RS20760 | - | - | - | ABAYE_RS01395 | PAU_RS21685 |
| TAUDO - Taurine dioxygenase | b0368 | - | - | - | APECO1_RS01890 | - | B21_00322 | - | ABAYE_RS11215 | PAU_RS06080 |
| GMHEPAT - D-glycero-D-manno-hepose 1-phosphate adenyltransferase | b3052 | USA300HOU_RS03470 | - | Gmet_0922 | APECO1_RS17765 | - | B21_02872 | - | - | PAU_RS17530 |
| S7PI - Sedoheptulose 7-phosphate isomerase | b0222 | USA300HOU_RS01685 | - | Gmet_0920 | APECO1_1745 | - | B21_00221 | - | - | PAU_RS16170 |
| GMHEPPA - D-glycero-D-manno-heptose 1,7-bisphosphate phosphatase | b0200 | USA300HOU_RS02915 | - | Gmet_0923 | APECO1_RS01055 | - | B21_00198 | - | - | PAU_RS03320 |
| OMMBLHXy - 2-Octaprenyl-3-methyl-6-methoxy-1,4-benzoquinol hydroxylase (NADPHl) | b0662 | - | - | - | - | - | - | - | - | PAU_RS15550 |
| 2S6HCCi - 2 succinyl 6 hydroxy 2 4 cyclohexadiene 1 carboxylate synthase | - | - | - | - | - | - | - | BSU30820 | - | PAU_RS07500 |
| HADPCOADH3 - 3-hydroxyadipyl-CoA dehydrogenase (NAD+) | b1395 | - | - | - | - | - | - | - | - | PAU_RS11830 |
| FALDH2 - Formaldehyde dehydrogenase | b0356 | - | - | - | APECO1_RS01845 | - | B21_00314 | - | - | PAU_RS19365 |
| PMDPHT - Pyrimidine phosphatase | - | USA300HOU_RS09395 and USA300HOU_RS09385 | - | - | - | - | - | - | - | PAU_RS09280 |
| HEMEti - Heme transport via ABC system | - | (USA300HOU_RS05645 and USA300HOU_RS05640 and USA300HOU_RS05650 and USA300HOU_RS05655 and USA300HOU_RS05660 and USA300HOU_RS05665) or (USA300HOU_RS05645 and USA300HOU_RS05640 and USA300HOU_RS05650 and USA300HOU_RS09190 and USA300HOU_RS11775 and USA300HOU_RS11780) | - | - | - | - | - | - | - | PAU_RS13005 |
| SPMDabc - Spermidine transport via ABC system | (b1443 and b1442 and b1440 and b1441) or (b1123 and b1124 and b1125 and b1126) | USA300HOU_RS05500 and USA300HOU_RS05495 and USA300HOU_RS05490 and USA300HOU_RS05485 | - | - | - | CD630_10240 or CD630_10260 | - | - | - | PAU_RS03785 |
| FE3abc - Iron (III) transport via ABC system | - | - | (Mbar_A2262 or Mbar_A2397 or Mbar_A2394 or Mbar_A2267 or Mbar_A1172) and (Mbar_A2398 or Mbar_A1173 or Mbar_A2261 or Mbar_A2393) and (Mbar_A2263 or Mbar_A2395 or Mbar_A1174 or Mbar_A2396) | - | - | CD630_35270 and CD630_35300 | - | - | - | PAU_RS06790 |
| GLCpts - D-glucose transport via PEP:Pyr PTS | (b2417 and b1101 and b2415 and b2416) or (b1817 and b1818 and b1819 and b2415 and b2416) or (b2417 and b1621 and b2415 and b2416) | USA300HOU_RS05395 and USA300HOU_RS05400 and USA300HOU_RS01000 and ((USA300HOU_RS13765 and USA300HOU_RS12210) or USA300HOU_RS01255) | - | - | - | CD630_03880 or CD630_31270 or CD630_31250 or CD630_30970 or (CD630_30300 and CD630_30270) | - | BSU13890 and BSU13900 and BSU13910 | - | PAU_RS15165 and PAU_RS06805 |
| RNDR1 - Ribonucleoside-diphosphate reductase (ADP) | (b2582 and (b2234 and b2235)) or ((b2234 and b2235) and b3781) | USA300HOU_RS03950 and USA300HOU_RS03945 and USA300HOU_RS03955 | Mbar_A3615 | Gmet_1297 | ((APECO1_RS12465 and APECO1_RS12470) and APECO1_RS14600) or ((APECO1_RS12465 and APECO1_RS12470) and trxA) | (CD630_29950 and CD630_29940) or CD630_12610 | ((B21_02119 and B21_02120) and B21_02440) or ((B21_02119 and B21_02120) and B21_03608) | BSU17380 and BSU17390 and BSU17370 | ABAYE_RS15285 and ABAYE_RS15295 | (PAU_RS07575 and PAU_RS07570) and PAU_RS20575 |
| RNTR1 - Ribonucleoside triphosphate reductase ATP | b4238 | USA300HOU_RS14195 and USA300HOU_RS14200 | Mbar_A1037 | | - | - | - | - | - | PAU_RS19855 |
| RNDR2 - Ribonucleoside-diphosphate reductase (GDP) | ((b2235 and b2234) and b2582) or ((b2235 and b2234) and b3781) | USA300HOU_RS03950 and USA300HOU_RS03945 and USA300HOU_RS03955 | Mbar_A3615 | Gmet_1297 | - | (CD630_29950 and CD630_29940) or CD630_12610 | - | BSU17380 and BSU17390 and BSU17370 | ABAYE_RS15285 and ABAYE_RS15295 | (PAU_RS07575 and PAU_RS07570) and PAU_RS20575 |
| RNTR2 - Ribonucleoside triphosphate reductase GTP | b4238 | USA300HOU_RS14195 and USA300HOU_RS14200 | Mbar_A1037 | - | - | - | - | - | - | PAU_RS19855 |
| RNTR3 - Ribonucleoside triphosphate reductase CTP | b4238 | USA300HOU_RS14195 and USA300HOU_RS14200 | Mbar_A1037 | - | - | - | - | - | - | PAU_RS19855 |
| RNTR4 - Ribonucleoside triphosphate reductase UTP | b4238 | USA300HOU_RS14195 and USA300HOU_RS14200 | Mbar_A1037 | - | - | - | - | - | - | PAU_RS19855 |
| PAPSR - Phosphoadenylyl-sulfate reductase (thioredoxin) | (b2762 and b3781) or (b2762 and b2582) | - | Mbar_A0559 or Mbar_A2943 | - | (APECO1_RS15670 and APECO1_RS14600) or (APECO1_RS15670 and trxA) | - | (B21_02570 and B21_02440) or (B21_02570 and B21_03608) | - | ABAYE_RS04095 | (PAU_RS03390 and PAU_RS20575) |
| ILEt2r - L isoleucine reversible transport via proton symport | b0401 | USA300HOU_RS00980 or USA300HOU_RS01625 | Mbar_A2616 and Mbar_A2617 | - | - | - | - | BSU26690 or (BSU26710 and BSU26700) or BSU29600 | ABAYE_RS08425 | PAU_RS17320 |
| MECDPDH2 - 2C-methyl-D-erythritol 2,4 cyclodiphosphate dehydratase | b2515 | - | - | - | APECO1_RS14275 | - | B21_02369 | BSU25070 | ABAYE_RS16240 | PAU_RS06720 |
| HISt2r - L histidine reversible transport via proton symport | b0112 | - | - | - | - | - | - | BSU39390 or BSU02400 | - | PAU_RS08465 |
| H2Ot - H2O transport via diffusion | b0875 | - | - | - | - | - | - | - | - | PAU_RS12365 |
| 3HAD140 - 3-hydroxyacyl-[acyl-carrier-protein] dehydratase (n-C14:0) | b0180 or b0954 | USA300HOU_RS11335 | - | Gmet_2354 or Gmet_1697 | APECO1_59 or APECO1_RS00950 | - | - | - | - | PAU_RS03240 or PAU_RS13340 |
| 4M3OXHA - 4-methyl-3-oxo-hexanoyl-ACP NADP oxidoreductase | - | USA300HOU_RS06185 | - | - | - | - | - | - | - | PAU_RS08255 |
| 4ISOHELS - 4-methyl-3-hydroxy-hexanoyl-ACP hydro-lyase | - | USA300HOU_RS11335 | - | - | - | - | - | - | - | PAU_RS03240 |
| 6M3OXO - 6-methyl-3-oxo-octanoyl-ACP NADP oxidoreductase | - | USA300HOU_RS06185 | - | - | - | - | - | - | - | PAU_RS08255 |
| 6M3HOAHL - 6-methyl-3-hydroxy-octanoyl-ACP hydro-lyase | - | USA300HOU_RS11335 | - | - | - | - | - | - | - | PAU_RS03240 |
| 8M3OAO - 8-methyl-3-oxo-decanoyl-ACP NADP oxidoreductase | - | USA300HOU_RS06185 | - | - | - | - | - | - | - | PAU_RS08255 |
| 8M3HDAL - 8-methyl-3-hydroxy-decanoyl-ACP hydro-lyase | - | USA300HOU_RS11335 | - | - | - | - | - | - | - | PAU_RS03240 |
| 10M3OACPO - 10-methyl-3-oxo-dodecanoyl-ACP NADP oxidoreductase | - | USA300HOU_RS06185 | - | - | - | - | - | - | - | PAU_RS08255 |
| 2MPCTF - 10-methyl-3-hydroxy-dodecanoyl-ACP hydro-lyase | - | USA300HOU_RS11335 | - | - | - | - | - | - | - | PAU_RS03240 |
| 12M3TDA - 12-methyl-3-oxo-tetra-decanoyl-ACP NADP oxidoreductase | - | USA300HOU_RS06185 | - | - | - | - | - | - | - | PAU_RS08255 |
| 12M3HTDH - 12-methyl-3-hydroxy-tetra-decanoyl-ACP hydro-lyase | - | USA300HOU_RS11335 | - | - | - | - | - | - | - | PAU_RS03240 |
| 14M3OHO - 14-methyl-3-oxo-hexa-decanoyl-ACP NADP oxidoreductase | - | USA300HOU_RS06185 | - | - | - | - | - | - | - | PAU_RS08255 |
| 14M3HDEC - 14-methyl-3-hydroxy-hexa-decanoyl-ACP hydro-lyase | - | USA300HOU_RS11335 | - | - | - | - | - | - | - | PAU_RS03240 |
| 5MET3OH - 5-methyl-3-oxo-hexanoyl-ACP NADP oxidoreductase | - | USA300HOU_RS06185 | - | - | - | - | - | - | - | PAU_RS08255 |
| 5M3HHAL - 5-methyl-3-hydroxy-hexanoyl-ACP hydro-lyase | - | USA300HOU_RS11335 | - | - | - | - | - | - | - | PAU_RS03240 |
| 7M3ODO - 7-methyl-3-oxo-octanoyl-ACP NADP oxidoreductase | - | USA300HOU_RS06185 | - | - | - | - | - | - | - | PAU_RS08255 |
| 7M3HOACPL - 7-methyl-3-hydroxy-octanoyl-ACP hydro-lyase | - | USA300HOU_RS11335 | - | - | - | - | - | - | - | PAU_RS03240 |
| 9M3OXPA - 9-methyl-3-oxo-decanoyl-ACP NADP oxidoreductase | - | USA300HOU_RS06185 | - | - | - | - | - | - | - | PAU_RS08255 |
| 9M3HDL - 9-methyl-3-hydroxy-decanoyl-ACP hydro-lyase | - | USA300HOU_RS11335 | - | - | - | - | - | - | - | PAU_RS03240 |
| 11M3ODO - 11-methyl-3-oxo-dodecanoyl-ACP NADP oxidoreductase | - | USA300HOU_RS06185 | - | - | - | - | - | - | - | PAU_RS08255 |
| 11MHDOD - 11-methyl-3-hydroxy-dodecanoyl-ACP hydro-lyase | - | USA300HOU_RS11335 | - | - | - | - | - | - | - | PAU_RS03240 |
| 13M3OTDAO - 13-methyl-3-oxo-tetra-decanoyl-ACP NADP oxidoreductase | - | USA300HOU_RS06185 | - | - | - | - | - | - | - | PAU_RS08255 |
| 13M3HTDAHL - 13-methyl-3-hydroxy-tetra-decanoyl-ACP hydro-lyase | - | USA300HOU_RS11335 | - | - | - | - | - | - | - | PAU_RS03240 |
| 15M3OHAO - 15-methyl-3-oxo-hexa-decanoyl-ACP NADP oxidoreductase | - | USA300HOU_RS06185 | - | - | - | - | - | - | - | PAU_RS08255 |
| 15M3HEXDA - 15-methyl-3-hydroxy-hexa-decanoyl-ACP hydro-lyase | - | USA300HOU_RS11335 | - | - | - | - | - | - | - | PAU_RS03240 |
| ACGApts - N-Acetyl-D-glucosamine transport via PEP:Pyr PTS | - | USA300HOU_RS05400 and USA300HOU_RS01375 and USA300HOU_RS01000 and USA300HOU_RS09160 and USA300HOU_RS05395 | - | - | - | CD630_31370 | - | BSU07700 and BSU13900 and BSU13910 | - | PAU_RS06805 and PAU_RS15165 |
| ARBTpts - Arbutin transport via PEPPyr PTS | - | USA300HOU_RS05395 and USA300HOU_RS05400 | - | - | - | CD630_26660 and CD630_27550 | - | BSU39270 and BSU13900 and BSU13910 | - | PAU_RS06810 and PAU_RS06805 |
| CAt4 - Calcium transport inout via proton antiporter | - | - | - |  | - | - | - | BSU07920 | - | PAU_RS18210 |
| CELBpts - Cellobiose transport via PEPPyr PTS | - | - | - | - | - | CD630_27550 and CD630_36480 and CD630_36470 | - | (BSU13900 and BSU13910 and BSU38390) or (BSU13900 and BSU13910 and BSU05810 and BSU05820 and BSU05830) or (BSU38570 and BSU38590 and BSU38580 and BSU13900 and BSU13910) | - | PAU_RS06805 and PAU_RS08625 and PAU_RS08615 |
| FRUpts - D-fructose transport via PEP:Pyr PTS | - | USA300HOU_RS05395 and USA300HOU_RS05400 and USA300HOU_RS03780 | - | - | - | CD630_31340 or (CD630_02060 and CD630_02070 and CD630_02080) or (CD630_32780 and CD630_32790 and CD630_32770 and CD630_32760) or (CD630_10740 and CD630_10760 and CD630_10770 and CD630_10780) or (CD630_04910 and CD630_04920 and CD630_04930 and CD630_04940) or (CD630_02860 and CD630_02870 and CD630_02880 and CD630_02890) or (CD630_02840 and CD630_02850) or (CD630_36290 and CD630_36300) | - | (BSU27070 and BSU27060 and BSU27050 and BSU27040 and BSU13900 and BSU13910) or (BSU14400 and BSU13900 and BSU13910) | - | PAU_RS06810 and PAU_RS06805 and PAU_RS18250 |
| GALTpts - Galactitol transport via PEP:Pyr PTS | b2415 and b2092 and b2094 and b2416 and b2093 | USA300HOU_RS01275 and USA300HOU_RS01280 and USA300HOU_RS01285 and USA300HOU_RS05395 and USA300HOU_RS05400 | - | - | - | (CD630_00430 and CD630_00420 and CD630_00410) or (CD630_10830 and CD630_22800 and CD630_22820) or (CD630_23270 and CD630_23260 and CD630_23250) | - | - | - | PAU_RS06810 and PAU_RS06805 |
| GAMpts - D-glucosamine transport via PEP:Pyr PTS | b2415 and b2416 and b1818 and b1817 and b1819 | USA300HOU_RS05395 and USA300HOU_RS05400 | - | - | - | CD630_04690 or CD630_08160 or CD630_30970 | - | BSU02350 and BSU13900 and BSU13910 | - | PAU_RS06810 and PAU_RS06805 |
| MALTTRabc - Maltotriose transport via ABC system | b4032 and b4035 and b4033 and b4036 and b4034 | USA300HOU_RS01095 and USA300HOU_RS01090 and USA300HOU_RS01105 and USA300HOU_RS01100 | - | - | - | - | - | - | - | PAU_RS01825 and PAU_RS01810 and PAU_RS01820 and PAU_RS01805 and PAU_RS01815 |
| MANpts - D-mannose transport via PEP:Pyr PTS | b2415 and b2416 and b1818 and b1819 and b1817 | USA300HOU_RS14355 and USA300HOU_RS05400 and USA300HOU_RS01000 and USA300HOU_RS05395 | - | - | - | (CD630_30150 and CD630_30140 and CD630_30130) or (CD630_25660 and CD630_25670 and CD630_25680) or (CD630_32780 and CD630_32790 and CD630_32770 and CD630_32760) or (CD630_10740 and CD630_10760 and CD630_10770 and CD630_10780) or (CD630_04910 and CD630_04920 and CD630_04930 and CD630_04940) or (CD630_02860 and CD630_02870 and CD630_02880 and CD630_02890) or (CD630_02840 and CD630_02850) | - | BSU12010 and BSU13900 and BSU13910 | - | PAU_RS06810 and PAU_RS06805 and PAU_RS08855 and PAU_RS08850 and PAU_RS08860 |
| MNLpts - Mannitol transport via PEP:Pyr PTS | b2415 and b2416 and b3599 | USA300HOU_RS11635 and USA300HOU_RS11640 and USA300HOU_RS11630 and USA300HOU_RS05395 and USA300HOU_RS05400 | - | - | - | CD630_23320 and CD630_23340 | - | BSU03981 and BSU13900 and BSU13910 | - | PAU_RS06810 and PAU_RS06805 |
| SALCpts - Salicin transport via PEP:Pyr PTS | - | USA300HOU_RS05395 and USA300HOU_RS05400 | - | - | - | CD630_26660 and CD630_27550 | - | BSU39270 and BSU13900 and BSU13910 | - | PAU_RS06810 and PAU_RS06805 |
| SUCpts - Sucrose transport via PEP:Pyr PTS | b2415 and b2417 and b2429 and b2416 | USA300HOU_RS12870 and USA300HOU_RS05395 and USA300HOU_RS05400 | - | - | - | CD630_04690 | - | (BSU13900 and BSU13910 and BSU38050) or (BSU13900 and BSU13910 and BSU01680) | - | PAU_RS06810 and PAU_RS06800 and PAU_RS01560 and PAU_RS06805 |
| RNDR4 - Ribonucleoside-diphosphate reductase (UDP) | (b3781 and (b2234 and b2235)) or ((b2234 and b2235) and b2582) | USA300HOU_RS03950 and USA300HOU_RS03945 and USA300HOU_RS03955 | Mbar_A3615 | Gmet_1297 | - | (CD630_29950 and CD630_29940) or CD630_12610 | | BSU17380 and BSU17390 and BSU17370 | ABAYE_RS15285 and ABAYE_RS15295 | PAU_RS15695 and PAU_RS15690 and PAU_RS15700 |
| RNDR3 - Ribonucleoside-diphosphate reductase (CDP) | ((b2234 and b2235) and b3781) or (b2582 and (b2234 and b2235)) | USA300HOU_RS03950 and USA300HOU_RS03945 and USA300HOU_RS03955 | Mbar_A3615 | Gmet_1297 | - | (CD630_29950 and CD630_29940) or CD630_12610 | | BSU17380 and BSU17390 and BSU17370 | ABAYE_RS15285 and ABAYE_RS15295 | PAU_RS15695 and PAU_RS15690 and PAU_RS15700 |
| HACD6i - 3-hydroxyacyl-CoA dehydrogenase (3-oxotetradecanoyl-CoA) | b3846 or b2341 | - | - | - | APECO1_RS21985 or APECO1_RS12990 | - | B21_03686 or B21_02226 | - | - | PAU_RS19205 or PAU_RS06925 |
| AKGDH - 2-Oxogluterate dehydrogenase | b0726 and b0116 and b0727 | USA300HOU_RS07205 and USA300HOU_RS07210 | - | (Gmet_1896 and Gmet_2769 and Gmet_2766) or (Gmet_2764 and Gmet_2769 and Gmet_2766) | APECO1_RS00640 and APECO1_RS03495 and APECO1_RS03500 | CD630_07230 and CD630_00390 | B21_00114 and B21_00674 and B21_00675 | BSU19370 and BSU19360 and BSU14610 | - | PAU_RS14540 and PAU_RS05585 and PAU_RS14535 |
| AMALT1 - Amylomaltase (maltotriose) | b3416 | - | - | Gmet_2391 | APECO1_RS19540 | - | B21_03220 | - | - | PAU_RS01855 |
| AMALT2 - Amylomaltase (maltotetraose) | b3416 | - | - | Gmet_2391 | APECO1_RS19540 | - | B21_03220 | - | - | PAU_RS01855 |
| AMALT3 - Amylomaltase (maltopentaose) | b3416 | - | - | Gmet_2391 | APECO1_RS19540 | - | B21_03220 | - | - | PAU_RS01855 |
| AMALT4 - Amylomaltase (maltohexaose) | b3416 | - | - | Gmet_2391 | APECO1_RS19540 | - | B21_03220 | - | - | PAU_RS01855 |
| DHNAOT4 - 1,4-dihydroxy-2-naphthoate octaprenyltransferase | b3930 | - | - | Gmet_3393 and Gmet_3392 | APECO1_RS22415 | - | B21_03764 | - | - | PAU_RS21180 |
| FRD2 - Fumarate reductase | b4154 and b4151 and b4153 and b4152 | - | - | - | APECO1_RS24160 and APECO1_RS24165 and APECO1_RS24170 and APECO1_RS24175 | - | B21_03985 and B21_03986 and B21_03987 and B21_03988 | - | ABAYE_RS04415 and ABAYE_RS04420 and ABAYE_RS04425 and ABAYE_RS04430 | PAU_RS18575 and PAU_RS18590 and PAU_RS18580 and PAU_RS18585 |
| FRD3 - Fumarate reductase | b4154 and b4153 and b4151 and b4152 | - | - | - | APECO1_RS24160 and APECO1_RS24165 and APECO1_RS24170 and APECO1_RS24175 | - | B21_03985 and B21_03986 and B21_03987 and B21_03988 | - | ABAYE_RS04415 and ABAYE_RS04420 and ABAYE_RS04425 and ABAYE_RS04430 | PAU_RS18575 and PAU_RS18590 and PAU_RS18580 and PAU_RS18585 |
| HEPT1 - Heptosyltransferase I (LPS core synthesis) | b3621 | - | - | Gmet_2331 | APECO1_RS20710 | - | B21_03429 | - | - | PAU_RS21660 |
| HEPT3 - Heptosyltransferase III (LPS core synthesis) | b3632 | - | - | - | APECO1_RS20755 | - | B21_03441 | - | - | PAU_RS21670 |
| LPLIPAL2A120 - Lysophospholipase L2 (2-acylglycerophosphotidate, n-C12:0) | b3825 | - | - | - | APECO1_RS21770 | - | B21_03653 | - | - | PAU_RS20405 |
| LPLIPAL2A140 - Lysophospholipase L2 (2-acylglycerophosphotidate, n-C14:0) | b3825 | - | - | - | APECO1_RS21770 | - | B21_03653 | - | - | PAU_RS20405 |
| LPLIPAL2A141 - Lysophospholipase L2 (2-acylglycerophosphotidate, n-C14:1) | b3825 | - | - | - | APECO1_RS21770 | - | B21_03653 | - | - | PAU_RS20405 |
| LPLIPAL2A160 - Lysophospholipase L2 (2-acylglycerophosphotidate, n-C16:0) | b3825 | - | - | - | APECO1_RS21770 | - | B21_03653 | - | - | PAU_RS20405 |
| LPLIPAL2A161 - Lysophospholipase L2 (2-acylglycerophosphotidate, n-C16:1) | b3825 | - | - | - | APECO1_RS21770 | - | B21_03653 | - | - | PAU_RS20405 |
| LPLIPAL2A180 - Lysophospholipase L2 (2-acylglycerophosphotidate, n-C18:0) | b3825 | - | - | - | APECO1_RS21770 | - | B21_03653 | - | - | PAU_RS20405 |
| LPLIPAL2A181 - Lysophospholipase L2 (2-acylglycerophosphotidate, n-C18:1) | b3825 | - | - | - | APECO1_RS21770 | - | B21_03653 | - | - | PAU_RS20405 |
| LPLIPAL2E120 - Lysophospholipase L2 (2-acylglycerophosphoethanolamine, n-C12:0) | b3825 | - | - | - | APECO1_RS21770 | - | B21_03653 | - | - | PAU_RS20405 |
| LPLIPAL2E140 - Lysophospholipase L2 (2-acylglycerophosphoethanolamine, n-C14:0) | b3825 | - | - | - | APECO1_RS21770 | - | B21_03653 | - | - | PAU_RS20405 |
| LPLIPAL2E141 - Lysophospholipase L2 (2-acylglycerophosphoethanolamine, n-C14:1) | b3825 | - | - | - | APECO1_RS21770 | - | B21_03653 | - | - | PAU_RS20405 |
| LPLIPAL2E160 - Lysophospholipase L2 (2-acylglycerophosphoethanolamine, n-C16:0) | b3825 | - | - | - | APECO1_RS21770 | - | B21_03653 | - | - | PAU_RS20405 |
| LPLIPAL2E161 - Lysophospholipase L2 (2-acylglycerophosphoethanolamine, n-C16:1) | b3825 | - | - | - | APECO1_RS21770 | - | B21_03653 | - | - | PAU_RS20405 |
| LPLIPAL2E180 - Lysophospholipase L2 (2-acylglycerophosphoethanolamine, n-C18:0) | b3825 | - | - | - | APECO1_RS21770 | - | B21_03653 | - | - | PAU_RS20405 |
| LPLIPAL2E181 - Lysophospholipase L2 (2-acylglycerophosphoethanolamine, n-C18:1) | b3825 | - | - | - | APECO1_RS21770 | - | B21_03653 | - | - | PAU_RS20405 |
| LPLIPAL2G120 - Lysophospholipase L2 (2-acylglycerophosphoglycerol, n-C12:0) | b3825 | - | - | - | APECO1_RS21770 | - | B21_03653 | - | - | PAU_RS20405 |
| LPLIPAL2G140 - Lysophospholipase L2 (2-acylglycerophosphoglycerol, n-C14:0) | b3825 | - | - | - | APECO1_RS21770 | - | B21_03653 | - | - | PAU_RS20405 |
| LPLIPAL2G141 - Lysophospholipase L2 (2-acylglycerophosphoglycerol, n-C14:1) | b3825 | - | - | - | APECO1_RS21770 | - | B21_03653 | - | - | PAU_RS20405 |
| LPLIPAL2G160 - Lysophospholipase L2 (2-acylglycerophosphoglycerol, n-C16:0) | b3825 | - | - | - | APECO1_RS21770 | - | B21_03653 | - | - | PAU_RS20405 |
| LPLIPAL2G161 - Lysophospholipase L2 (2-acylglycerophosphoglycerol, n-C16:1) | b3825 | - | - | - | APECO1_RS21770 | - | B21_03653 | - | - | PAU_RS20405 |
| LPLIPAL2G180 - Lysophospholipase L2 (2-acylglycerophosphoglycerol, n-C18:0) | b3825 | - | - | - | APECO1_RS21770 | - | B21_03653 | - | - | PAU_RS20405 |
| LPLIPAL2G181 - Lysophospholipase L2 (2-acylglycerophosphoglycerol, n-C18:1) | b3825 | - | - | - | APECO1_RS21770 | - | B21_03653 | - | - | PAU_RS20405 |
| LPLIPAL2A120 - Lysophospholipase L2 (2-acylglycerophosphotidate, n-C12:0) | b3825 | - | - | - | APECO1_RS21770 | - | B21_03653 | - | - | PAU_RS20405 |
| LPLIPAL2A140 - Lysophospholipase L2 (2-acylglycerophosphotidate, n-C14:0) | b3825 | - | - | - | APECO1_RS21770 | - | B21_03653 | - | - | PAU_RS20405 |
| LPLIPAL2A141 - Lysophospholipase L2 (2-acylglycerophosphotidate, n-C14:1) | b3825 | - | - | - | APECO1_RS21770 | - | B21_03653 | - | - | PAU_RS20405 |
| LPLIPAL2A160 - Lysophospholipase L2 (2-acylglycerophosphotidate, n-C16:0) | b3825 | - | - | - | APECO1_RS21770 | - | B21_03653 | - | - | PAU_RS20405 |
| LPLIPAL2A161 - Lysophospholipase L2 (2-acylglycerophosphotidate, n-C16:1) | b3825 | - | - | - | APECO1_RS21770 | - | B21_03653 | - | - | PAU_RS20405 |
| LPLIPAL2A180 - Lysophospholipase L2 (2-acylglycerophosphotidate, n-C18:0) | b3825 | - | - | - | APECO1_RS21770 | - | B21_03653 | - | - | PAU_RS20405 |
| LPLIPAL2A181 - Lysophospholipase L2 (2-acylglycerophosphotidate, n-C18:1) | b3825 | - | - | - | APECO1_RS21770 | - | B21_03653 | - | - | PAU_RS20405 |
| LPLIPAL2E120 - Lysophospholipase L2 (2-acylglycerophosphoethanolamine, n-C12:0) | b3825 | - | - | - | APECO1_RS21770 | - | B21_03653 | - | - | PAU_RS20405 |
| LPLIPAL2E140 - Lysophospholipase L2 (2-acylglycerophosphoethanolamine, n-C14:0) | b3825 | - | - | - | APECO1_RS21770 | - | B21_03653 | - | - | PAU_RS20405 |
| LPLIPAL2E141 - Lysophospholipase L2 (2-acylglycerophosphoethanolamine, n-C14:1) | b3825 | - | - | - | APECO1_RS21770 | - | B21_03653 | - | - | PAU_RS20405 |
| LPLIPAL2E160 - Lysophospholipase L2 (2-acylglycerophosphoethanolamine, n-C16:0) | b3825 | - | - | - | APECO1_RS21770 | - | B21_03653 | - | - | PAU_RS20405 |
| LPLIPAL2E161 - Lysophospholipase L2 (2-acylglycerophosphoethanolamine, n-C16:1) | b3825 | - | - | - | APECO1_RS21770 | - | B21_03653 | - | - | PAU_RS20405 |
| LPLIPAL2E180 - Lysophospholipase L2 (2-acylglycerophosphoethanolamine, n-C18:0) | b3825 | - | - | - | APECO1_RS21770 | - | B21_03653 | - | - | PAU_RS20405 |
| LPLIPAL2E181 - Lysophospholipase L2 (2-acylglycerophosphoethanolamine, n-C18:1) | b3825 | - | - | - | APECO1_RS21770 | - | B21_03653 | - | - | PAU_RS20405 |
| LPLIPAL2G120 - Lysophospholipase L2 (2-acylglycerophosphoglycerol, n-C12:0) | b3825 | - | - | - | APECO1_RS21770 | - | B21_03653 | - | - | PAU_RS20405 |
| LPLIPAL2G140 - Lysophospholipase L2 (2-acylglycerophosphoglycerol, n-C14:0) | b3825 | - | - | - | APECO1_RS21770 | - | B21_03653 | - | - | PAU_RS20405 |
| LPLIPAL2G141 - Lysophospholipase L2 (2-acylglycerophosphoglycerol, n-C14:1) | b3825 | - | - | - | APECO1_RS21770 | - | B21_03653 | - | - | PAU_RS20405 |
| LPLIPAL2G160 - Lysophospholipase L2 (2-acylglycerophosphoglycerol, n-C16:0) | b3825 | - | - | - | APECO1_RS21770 | - | B21_03653 | - | - | PAU_RS20405 |
| LPLIPAL2G161 - Lysophospholipase L2 (2-acylglycerophosphoglycerol, n-C16:1) | b3825 | - | - | - | APECO1_RS21770 | - | B21_03653 | - | - | PAU_RS20405 |
| LPLIPAL2G180 - Lysophospholipase L2 (2-acylglycerophosphoglycerol, n-C18:0) | b3825 | - | - | - | APECO1_RS21770 | - | B21_03653 | - | - | PAU_RS20405 |
| LPLIPAL2G181 - Lysophospholipase L2 (2-acylglycerophosphoglycerol, n-C18:1) | b3825 | - | - | - | APECO1_RS21770 | - | B21_03653 | - | - | PAU_RS20405 |
| MLTP1 - Maltodextrin phosphorylase (maltopentaose) | b3417 | - | - | - | APECO1_RS19545 | - | B21_03221 | - | - | PAU_RS01860 |
| MLTP2 - Maltodextrin phosphorylase (maltohexaose) | b3417 | - | - | - | APECO1_RS19545 | - | B21_03221 | - | - | PAU_RS01860 |
| MLTP3 - Maltodextrin phosphorylase (maltoheptaose) | b3417 | - | - | - | APECO1_RS19545 | - | B21_03221 | - | - | PAU_RS01860 |
| PGPP120 - Phosphatidylglycerol phosphate phosphatase (n-C14:0) | b0418 or b1278 | - | - | Gmet_0195 | APECO1_RS07090 or APECO1_RS02125 | - | B21_01266 or B21_00370 | - | - | PAU_RS17235 or PAU_RS24470 |
| PGPP140 - Phosphatidylglycerol phosphate phosphatase (n-C14:0) | b0418 or b1278 | - | - | Gmet_0195 | APECO1_RS07090 or APECO1_RS02125 | - | B21_01266 or B21_00370 | - | - | PAU_RS17235 or PAU_RS24470 |
| PGPP141 - Phosphatidylglycerol phosphate phosphatase (n-C14:1) | b0418 or b1278 | - | - | Gmet_0195 | APECO1_RS07090 or APECO1_RS02125 | - | B21_01266 or B21_00370 | - | - | PAU_RS17235 or PAU_RS24470 |
| PGPP160 - Phosphatidylglycerol phosphate phosphatase (n-C16:0) | b0418 or b1278 | - | - | Gmet_0195 | APECO1_RS07090 or APECO1_RS02125 | - | B21_01266 or B21_00370 | - | - | PAU_RS17235 or PAU_RS24470 |
| PGPP161 - Phosphatidylglycerol phosphate phosphatase (n-C16:1) | b0418 or b1278 | - | - | Gmet_0195 | APECO1_RS07090 or APECO1_RS02125 | - | B21_01266 or B21_00370 | - | - | PAU_RS17235 or PAU_RS24470 |
| PGPP180 - Phosphatidylglycerol phosphate phosphatase (n-C18:0) | b0418 or b1278 | - | - | Gmet_0195 | APECO1_RS07090 or APECO1_RS02125 | - | B21_01266 or B21_00370 | - | - | PAU_RS17235 or PAU_RS24470 |
| PGPP181 - Phosphatidylglycerol phosphate phosphatase (n-C18:1) | b0418 or b1278 | - | - | Gmet_0195 | APECO1_RS07090 or APECO1_RS02125 | - | B21_01266 or B21_00370 | - | - | PAU_RS17235 or PAU_RS24470 |
| PPPGO3 - Protoporphyrinogen oxidase (anaerobic) | b3850 | - | - | - | APECO1_RS22005 | - | B21_03690 | - | - | PAU_RS19185 |
| FRD7 - Fumarate reductase | b4151 and b4152 and b4153 and b4154 | - | - | - | - | - | - | - | - | PAU_RS18590 and PAU_RS18585 and PAU_RS18580 and PAU_RS18575 |
| THZPSN - Thiazole phosphate synthesis | b2530 and b3992 and b4407 and b0423 and (b3991 and b3990) | - | Mbar_A1238 and Mbar_A0010 | - | APECO1_RS14350 and APECO1_RS22770 and (APECO1_RS22755 and APECO1_RS22760) and APECO1_RS02150 and thiS | - | B21_02386 and B21_03822 and (B21_03819 and B21_03820) and B21_00375 and B21_03821 | - | - | PAU_RS06625 and PAU_RS01935 and PAU_RS01930 and PAU_RS01825 and (PAU_RS01925 and PAU_RS01920) |
| ATPS4r - ATP synthase (four protons for one ATP) | ((b3736 and b3737 and b3738) and (b3731 and b3732 and b3733 and b3734 and b3735)) or ((b3736 and b3737 and b3738) and (b3731 and b3732 and b3733 and b3734 and b3735) and b3739) | - | Mbar_A0384 and Mbar_A0385 and Mbar_A0389 and (Mbar_A0391 or Mbar_A0379) and (Mbar_A3102 or Mbar_A0386) and (Mbar_A0390 or Mbar_A0378) and Mbar_A0387 and Mbar_A0392 and (Mbar_A3101 or Mbar_A0388) | - | - | (CD630_34700 and CD630_34740 and CD630_34720 and CD630_34710 and CD630_34690 and CD630_34680 and CD630_29570 and CD630_29600 and CD630_34760) or (CD630_34700 and CD630_34740 and CD630_34720 and CD630_34710 and CD630_34690 and CD630_34680 and CD630_34730 and CD630_29600 and CD630_34760 and CD630_02510) | - | BSU36880 and BSU36870 and BSU36860 and BSU36850 and BSU36840 and BSU36830 and BSU36820 and BSU36810 and BSU36800 | - | (PAU_RS00180 and PAU_RS00185 and PAU_RS00190) and (PAU_RS00155 and PAU_RS00160 and PAU_RS00165 and PAU_RS00170 and PAU_RS00175) |
| AMMQT8_2 - S-adenosylmethione:2-demethylmenaquinone methyltransferase | - | - | - | - | APECO1_RS21925 | - | - | - | - | PAU_RS19260 |
| CYTBO3 - Cytochrome oxidase bo3 (ubiquinol-8: 2.5 protons) | b0432 and b0431 and b0429 and b0430 | - | - | - | - | - | - | - | - | PAU_RS16975 and PAU_RS16980 and PAU_RS16990 and PAU_RS16985 |
| FDH2 - Formate dehydrogenase (quinone-8: 2 protons) | (b1476 and b1474 and b1475) or b4079 or (b3892 and b3894 and b3893) | - | - | - | - | - | - | - | - | PAU_RS03685 and PAU_RS21830 and PAU_RS03690 |
| THD2 - NAD(P) transhydrogenase | b1602 and b1603 | - | - | - | - | - | - | - | ABAYE_RS06180 | PAU_RS11560 or PAU_RS11565 |
| CBL1abc - Cob(1)alamin transport via ABC system | b1709 and b0158 and b3966 and b1711 | - | - | - | - | - | - | - | - | PAU_RS09030 and PAU_RS04075 and PAU_RS09025 |
| SBTpts - D-sorbitol transport via PEP:Pyr PTS | b2703 and b2415 and b2702 and b2416 and b2704 | USA300HOU_RS05395 and USA300HOU_RS05400 | - | - | - | (CD630_07640 and CD630_07650 and CD630_07670) or (CD630_24140 and CD630_07650 and CD630_07670 and CD630_07660) | - | - | - | PAU_RS11860 and PAU_RS06810 and PAU_RS06805 |
| CHOLD - Choline dehydrogenase | - | USA300HOU_RS14155 | - | - | APECO1_RS01665 | CD630_30060 | B21_00270 | - | - | PAU_RS16745 |
| OMPHHX - 2-octaprenyl-6-methoxyphenol hydroxylase | b2907 | - | - | - | APECO1_RS16490 | - | B21_02702 | - | ABAYE_RS13405 | PAU_RS05665 |
| OPHHX - 2-Octaprenylphenol hydroxylase | b3835 | - | - | - | APECO1_RS21935 | - | B21_03677 | - | ABAYE_RS17030 | PAU_RS19250 |
| AMMQT7 - S adenosylmethione2 demthylmenaquinone methyltransferase menaquinone 7 | - | - | - | - | - | - | - | BSU22750 | - | PAU_RS19260 |

***Figure S1 –*** *Schematic representation of the metabolic network detailed in the genome-scale metabolic model, iEC879, of Photorhabdus asymbiotica. This metabolic map details the primary metabolic pathways and exchange reactions facilitating the survival of the bacteria. Obtained using the COBRA Toolbox in Matlab.*
