# Supplementary material for: A temperature-induced metabolic shift in the emerging human pathogen Photorhabdus asymbiotica
Source: mSystems. 2024 Oct 24;9(11):e00970-23. doi: 10.1128/msystems.00970-23 (PMC11575385; doi:10.1128/msystems.00970-23)
Supplement: File S6 — Further stoichiometric matrix analysis. [file msystems.00970-23-s0006.docx]

**Numerical Properties of iEC1073**

Numerical properties of the metabolic reconstruction of *P. asymbiotica* ATCC43949, iEC1073, was explored using the COBRA Toolbox v 3.0 (Heirendt et al. 2019) from within Matlab (R2019b). These properties provide an overview of the characteristics of the stoichiometric matrix detailing the reconstruction, and this analysis has also been performed on the draft reconstruction obtained from the Model SEED (Henry et al. 2010) to emphasize the necessity of manual curation to obtain a good quality genome-scale metabolic model.

**Stoichiometric Growth Equation**

The stoichiometric growth equation for iEC1073, was obtained using the *printRxnFormula* in the COBRA Toolbox v 3.0 implemented in Matlab (R2019b).

This growth equation is the same for the temperature-specific models and for biomass production in all *in silico* media conditions described in the study, the precursors and products of this reaction are not changed when simulating *in silico* growth in any condition.

***Reactants***

40.1102 atp_c[c] + 0.50987 gly_c[c] + 0.00309647 gthrd_c[c] + 0.00309647 thmpp_c[c] + 35.5403 h2o_c[c] + 0.200831 asp__L_c[c] + 0.00309647 amet_c[c] + 0.127801 met__L_c[c] + 0.00309647 nad_c[c] + 0.179456 ser__L_c[c] + 0.00309647 coa_c[c] + 0.219088 glu__L_c[c] + 0.0761465 cys__L_c[c] + 0.0160207 dctp_c[c] + 0.00309647 ACP_c[c] + DNArep_c[c] + 0.00309647 q8_c[c] + 0.135407 gtp_c[c] + 0.00309647 so4_c[c] + 0.0841036 ctp_c[c] + 0.00309647 10fthf_c[c] + 0.0908319 utp_c[c] + 0.00309647 ptrc_c[c] + 0.00309647 spmd_c[c] + 0.00309647 nadp_c[c] + 0.00309647 fad_c[c] + 0.00309647 5mthf_c[c] + 0.0160207 datp_c[c] + 0.154519 phe__L_c[c] + 0.184355 pro__L_c[c] + 0.00309647 pydx5p_c[c] + 0.0792636 his__L_c[c] + 0.219088 gln__L_c[c] + 0.200831 asn__L_c[c] + 0.427934 ala__L_c[c] + 0.00309647 mqn8_c[c] + 0.00309647 2dmmq8_c[c] + 0.010648 pe180_c[c] + 0.00309647 cu2_c[c] + 0.00309647 thf_c[c] + 0.246697 arg__L_c[c] + 0.00309647 ribflv_c[c] + 0.0160207 dttp_c[c] + 0.0160207 dgtp_c[c] + 0.120677 tyr__L_c[c] + 0.241799 ile__L_c[c] + 0.00309647 zn2_c[c] + 0.285438 lys__L_c[c] + 0.211073 thr__L_c[c] + RNAtranscrip_c[c] + 0.0472019 trp__L_c[c] + 0.352233 val__L_c[c] + 0.375389 leu__L_c[c] + protbiosyn_c[c] + 0.010648 phosglcdioctdec_c[c] + 0.010648 diiheppgly_c[c] + 0.00309647 fe3_c[c] + 0.00309647 mg2_c[c] + 0.00309647 k_c[c] + 0.00309647 cl_c[c] + 0.00309647 cobalt2_c[c] + 0.010648 diaiheppgly_c[c] + 0.00309647 adocbl_c[c] + 0.010648 dianethal_c[c] + 0.00309647 mn2_c[c] + 0.0250106 udcpdp_c[c] + 0.0250106 peptidopolyn_c[c] + 0.010648 didianethal_c[c] + 0.00309647 fe2_c[c] + 0.00309647 pheme_c[c] + 0.010648 antcard_c[c] + 0.010648 ihepcardlipin_c[c] + 0.0250106 colipa_c[c] + 0.010648 strcdlpn_c[c] + 0.00309647 sheme_c[c] + 0.00309647 ca2_c[c] + 1 photon_c[c] **->**

***Products***

0.484601 ppi_c[c] + 40 h_c[c] + 40 adp_c[c] + 39.9969 pi_c[c] + 0.00309647 HC01161_c[c] + 0.00309647 cbi_c[c] + 0.00309647 dmbzid_c[c] + 0.0250106 peptidopolyn1_c[c] + biomass_c[c]

**Basic Numerical Characteristics**

The number of elements (number of reactions multiplied by the number of metabolites) of the stoichiometric matrix in both iEC1073 and the draft reconstruction is detailed below **(Tab. 1)** and was obtained using the *size* function whilst the number of non-zero elements was obtained from the *nnz* function (Heirendt et al. 2019).

**Table 1. Size of the stoichiometric matrix in the draft and final metabolic reconstruction of *P. asymbiotica*.** The draft reconstruction of *P. asymbiotica* contains 1489 reaction, 1501 metabolites and 794 genes resulting in 2230486 elements in the stoichiometric matrix. Following gap filling, the number of reactions, metabolites and genes in the curated reconstruction (iEC1073) increases to 2033, 1770 and 1073 resulting in 3598410 elements in the stoichiometric matrix.

|  | **Draft Reconstruction** | **iEC1073** |
| --- | --- | --- |
| **Number of Reactions** | 1486 | 2033 |
| **Number of Metabolites** | 1501 | 1770 |
| **Number of Genes** | 794 | 1073 |
| **Number of Elements** | 2230486 | 3598410 |
| **Number of Non-Zero Elements** | 6646 | 9028 |

The sparsity of a matrix refers to the proportion of zero elements in a matrix, and a decrease in the sparsity of a stoichiometric matrix for a metabolic reconstruction. The sparsity ratio of the stoichiometric matrix for the draft reconstruction is 99.70% and 99.75% in iEC1073. Sparsity patterns of the stoichiometric matrices for the draft reconstruction and iEC1073 are displayed below **(Fig. 1)** and clearly show how following gap filling, the metabolites in the curated reconstruction are more interconnected and participate in more reactions in the network. These plots were generated following a tutorial available for the COBRA Toolbox (<https://opencobra.github.io/cobratoolbox/stable/tutorials/tutorialNumCharact.html>).



**Figure 1. Sparsity plots of the draft and curated reconstruction of iEC1073.** The manual gap filling performed during the network reconstruction results in far greater interconnectivity of metabolites in iEC1073, with more of the metabolites in the reconstruction taking part in a greater proportion of reactions. The colour denoted by the scale on the right of the plots describes the magnitude of the elements in the stoichiometric matrix involved in a particular reaction; the darker blue spot in both plots denotes the biomass reaction which utilizes the most metabolites. Plots generated in Matlab using the *spyc* algorithm, following the COBRA Toolbox tutorial (<https://opencobra.github.io/cobratoolbox/stable/tutorials/tutorialNumCharact.html>).

**Reaction and Metabolite Properties**

Flux variability analysis (FVA) was used to determine the minimum and maximum flux for each reaction in both the draft reconstruction and curated network to determine the number of blocked, essential and non-essential reactions. Blocked reactions refer to those which have both a minimum and maximum flux of zero, and are therefore inactive. Active reactions can be classified as essential, whereby both the minimum and maximum flux have a non-zero value, or non-essential where one of these fluxes is non-zero and the other zero. This analysis was performed using the *fluxVariability* algorithm (Heirendt et al. 2019) in unconstrained conditions with all exchange reactions open.

Though the proportion of blocked reactions is greater in iEC1073 compared to the draft reconstruction **(Tab. 2)**, this is likely due to the increase in the number of reactions being added to the model during gap filling. The most notable difference between the two networks is that the proportion of essential reactions in the curated version is decreased, indicating that during gap filling the number of alternative routes for the production of biomass precursors have been integrated with the network, as would be the case biologically.

|  |  | **% of Reactions** |  |
| --- | --- | --- | --- |
|  | **Blocked** | **Non-Essential** | **Essential** |
| **Draft Reconstruction** | 37.15 | 14.334 | 48.52 |
| **iEC1073** | 38.70 | 36.60 | 24.70 |

**Table 2. The proportion of active and inactive reactions in the draft and curated metabolic reconstruction of *P. asymbiotica*.** Utilizing Flux Variability Analysis (FVA) reactions in a metabolic network can be classified as inactive or blocked whereby both the minimum and maximum flux is zero or active where at least one of these reactions is non-zero. Active reactions can be further categorised as essential, with a non-zero maximum and minimum flux, or non-essential with one of these fluxes as zero. The proportion of blocked reactions across the two reconstructions is comparable, but the proportion of essential reactions is decreased in iEC1073 compared to the draft version, likely due to the addition of reactions during gap filling contributing to alternative routes for the synthesis of biomass precursors.

Similarly, the number of root metabolites which aren’t produced in the network were determined using *findRootNPMets* (Heirendt et al. 2019). iEC1073 contains 268 of these metabolites (15.14% of all metabolites) whilst this number is 75 (5% of all metabolites) in the draft reconstruction.

**Biomass Precursor Check**

Given that all analysis described in the manuscript involves optimizing for the biomass reaction (R_BIOMASS) as the objective function, the synthesis of biomass precursors in both reconstructions was tested using the *biomassPrecursorCheck* function (Heirendt et al. 2019).

Neither ACP nor a peptidoglycan polymer can be synthesized *in silico* in iEC1073 or the draft reconstruction, and the draft reconstruction is also not able to synthesize adenosylcobalamin *in silico*. However, both models can simulate biomass production *in silico* at rates of 199.43mmol/gDw/hr for the draft reconstruction and 141.42mmol/gDw/hr for iEC1073. The draft reconstruction is unable to grow *in silico* in minimal media conditions described in the manuscript, whilst iEC1073 produces biomass at a rate of 1.1076mmol/gDw/hr. Interestingly, in these constrained conditions iEC1073 is also unable to synthesize the biomass precursor adenosylcobalamin, like the draft reconstruction in unconstrained conditions.

**Topological Analysis**

The topology of the metabolic network was also investigated using the *networkTopology* algorithm (Heirendt et al. 2019) to determine metabolite connectivity, the number of reactions a metabolite participates in, and reaction length, the number of metabolites involved in a reaction.

The *testForTypeIIIPathways* function (Heirendt et al. 2019) confirmed that iEC1073 did not contain any loops, this is also true for the draft reconstruction. Network topology and the nullspace of the stoichiometric matrix can be found in Supplementary File 2.

**References**

[Heirendt, Laurent, Sylvain Arreckx, Thomas Pfau, Sebastián N. Mendoza, Anne Richelle, Almut Heinken, Hulda S. Haraldsdóttir, et al. 2019. “Creation and Analysis of Biochemical Constraint-Based Models Using the COBRA Toolbox v.3.0.” *Nature Protocols* 14 (3): 639–702.](http://paperpile.com/b/Q1KlM4/eCT4)

[Henry, Christopher S., Matthew DeJongh, Aaron A. Best, Paul M. Frybarger, Ben Linsay, and Rick L. Stevens. 2010. “High-Throughput Generation, Optimization and Analysis of Genome-Scale Metabolic Models.” *Nature Biotechnology* 28 (9): 977–82.](http://paperpile.com/b/Q1KlM4/p1Ii)
