# Supplementary material for: A temperature-induced metabolic shift in the emerging human pathogen Photorhabdus asymbiotica
Source: mSystems. 2024 Oct 24;9(11):e00970-23. doi: 10.1128/msystems.00970-23 (PMC11575385; doi:10.1128/msystems.00970-23)
Supplement: Supplemental Figures — Figures S1-S4. [file msystems.00970-23-s0007.docx]

**Supplementary Figure 1.** The proportion of active and inactive reactions, in constrained conditions, is less and greater than expected, respectively, when compared to five selected published prokaryotic GEMs [(Oh et al. 2007; Monk et al. 2017; Thiele et al. 2005; Kavvas et al. 2018; Charusanti et al. 2011)](https://paperpile.com/c/Q1KlM4/NrbR+jG6q+1GQg+MZR7+1NJy).
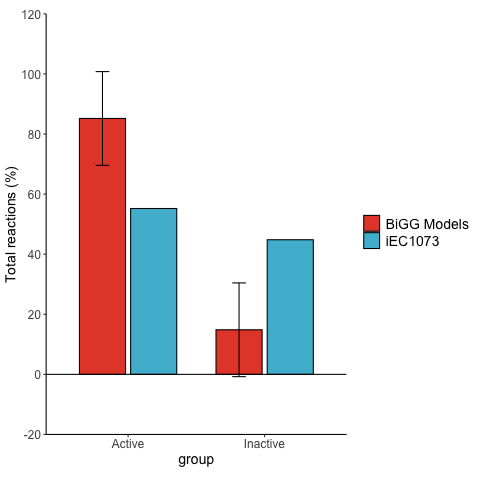


**Supplementary Figure 2.** Pathway distribution of genes in iEC1073 with a discordant essentiality status compared to that of orthologs in *E. coli*. The majority of these genes have an unannotated role whilst the remaining genes are relatively evenly distributed amongst the annotated metabolic functions, suggesting no major gaps remaining in the model requiring further curation.
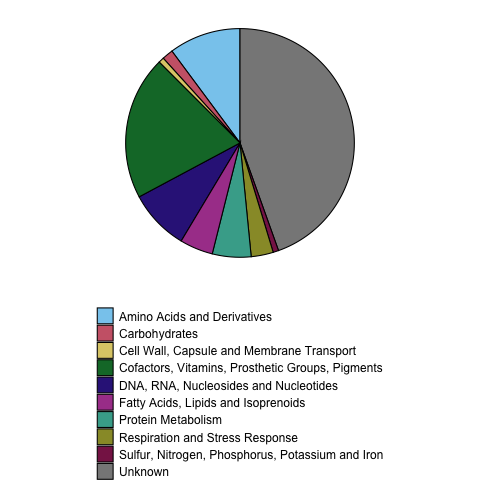


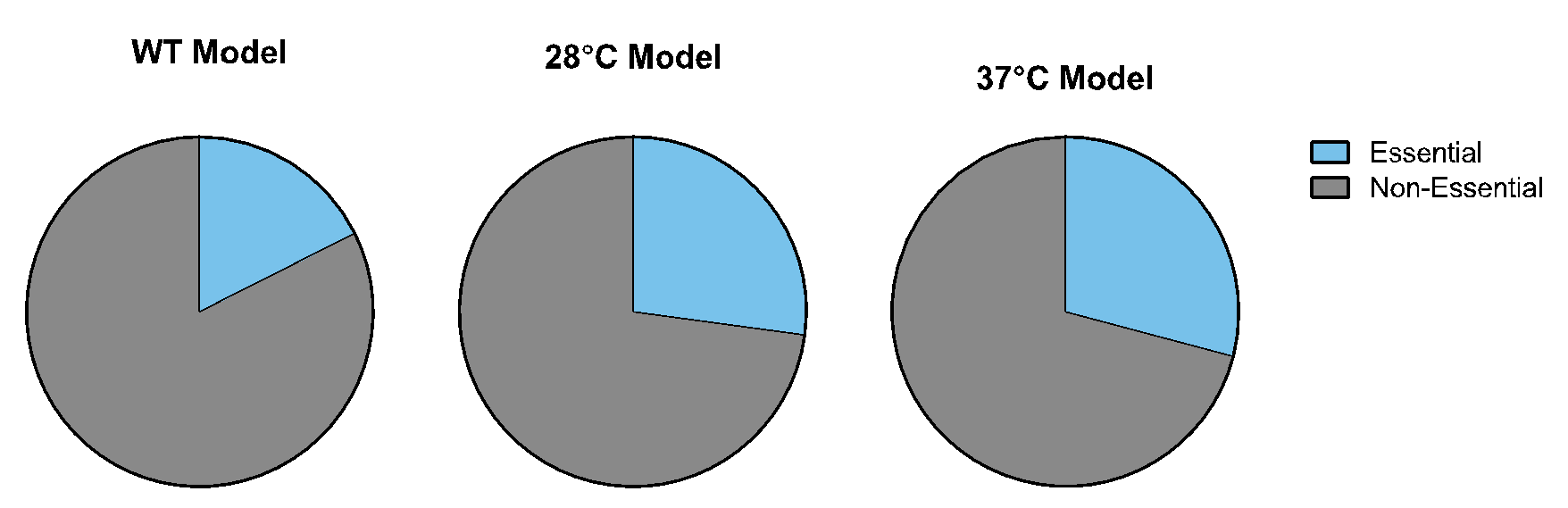


**Supplementary Figure 3.** Predicted gene essentiality in the wild-type metabolic reconstruction of *P. asymbiotica* compared to those predicted for the temperature-dependent reconstructions at 28°C and 37°C. At 28°C and 37°C, the number of essential genes increases to 291 and 311 respectively, from 194 in the wild-type reconstruction, respectively. Accordingly, the number of predicted non-essential genes at 28°C and 37°C decreases from 884 in the wild-type model to 782 and 762 at 28°C and 37°C, respectively.


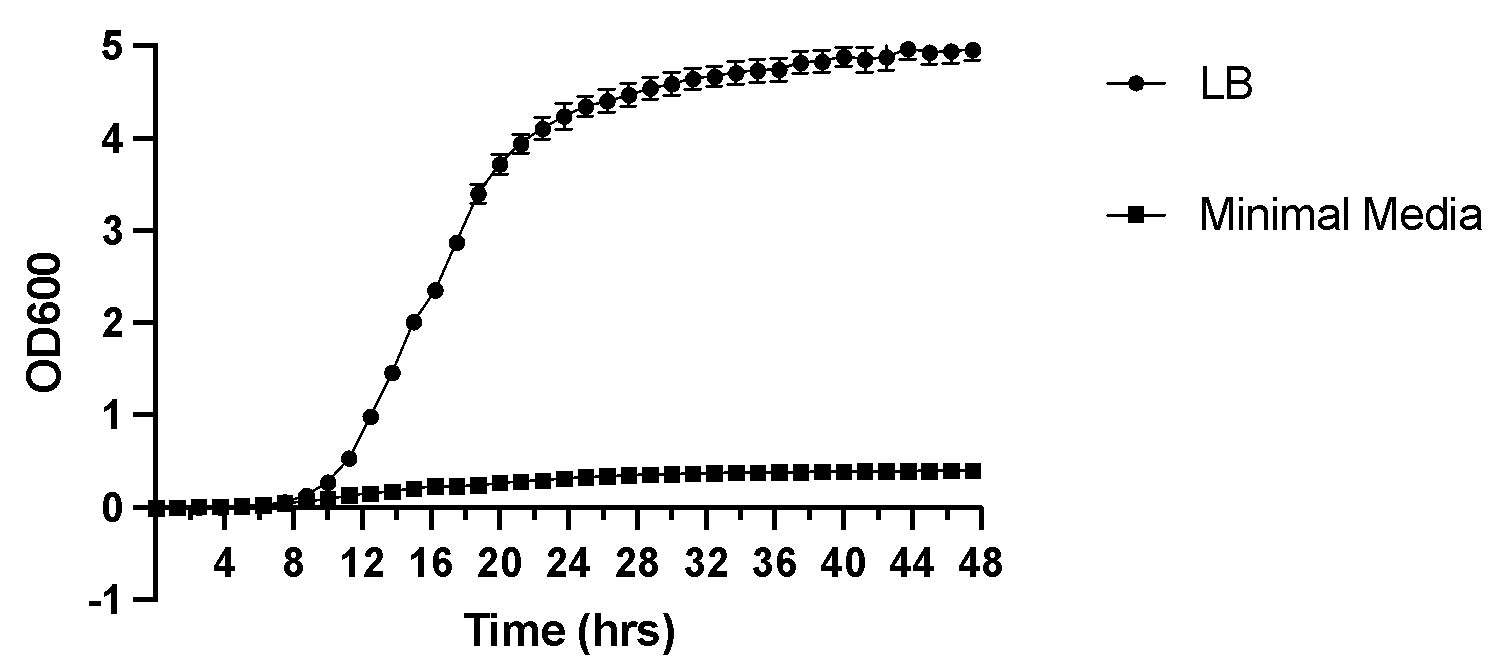


**Supplementary Figure 4.** Growth of *P. asymbiotica* ATCC43949 in LB and minimal media (M9 salts buffer, glucose, trace elements and casamino acids) over 48 hours at a temperature of 28°C. *P. asymbiotica* displays the typical exponential bacterial growth curve when grown in LB over 48 hours. In minimal media, the bacterium does not grow as well, as is expected in comparison with a rich medium such as LB, but OD600 does increase in these conditions over 48 hours.
